# Effect of Nitrogen Addition on Soil Microbial Functional Gene Abundance and Community Diversity in Permafrost Peatland

**DOI:** 10.3390/microorganisms9122498

**Published:** 2021-12-02

**Authors:** Xiuyan Ma, Yanyu Song, Changchun Song, Xianwei Wang, Nannan Wang, Siqi Gao, Xiaofeng Cheng, Zhendi Liu, Jinli Gao, Yu Du

**Affiliations:** 1 Key Laboratory of Wetland Ecology and Environment, Northeast Institute of Geography and Agroecology, Chinese Academy of Sciences, Changchun 130102, China; maxiuyan@iga.ac.cn (X.M.); songcc@iga.ac.cn (C.S.); wangxianwei@iga.ac.cn (X.W.); wangnannan@iga.ac.cn (N.W.); gaosiqi@iga.ac.cn (S.G.); c4x4yf@163.com (X.C.); liuzhendi20@mails.ucas.ac.cn (Z.L.); gaojinli@iga.ac.cn (J.G.); duyu@iga.ac.cn (Y.D.); 2 University of Chinese Academy Sciences, Beijing 100049, China; 3 Heilongjiang Province Key Laboratory of Geographical Environment Monitoring and Spatial Information Service in Cold Regions, Harbin Normal University, Harbin 150025, China

**Keywords:** nitrogen input, soil microbial functional gene abundance, soil microbial community diversity, permafrost peatland

## Abstract

Nitrogen is the limiting nutrient for plant growth in peatland ecosystems. Nitrogen addition significantly affects the plant biomass, diversity and community structure in peatlands. However, the response of belowground microbe to nitrogen addition in peatland ecosystems remains largely unknown. In this study, we performed long-term nitrogen addition experiments in a permafrost peatland in the northwest slope of the Great Xing’an Mountains. The four nitrogen addition treatments applied in this study were 0 g N·m^−2^·year^−1^ (CK), 6 g N·m^−2^·year^−1^ (N1), 12 g N·m^−2^·year^−1^ (N2), and 24 g N·m^−2^·year^−1^ (N3). Effects of nitrogen addition over a period of nine growing seasons on the soil microbial abundance and community diversity in permafrost peatland were analyzed. The results showed that the abundances of soil bacteria, fungi, archaea, nitrogen-cycling genes (*nif*H and b-*amo*A), and *mcr*A increased in N1, N2, and N3 treatments compared to CK. This indicated that nitrogen addition promoted microbial decomposition of soil organic matter, nitrogen fixation, ammonia oxidation, nitrification, and methane production. Moreover, nitrogen addition altered the microbial community composition. At the phylum level, the relative abundance of Proteobacteria increased significantly in the N2 treatment. However, the relative abundances of Actinobacteria and Verrucifera in the N2 treatment and Patescibacteria in the N1 treatment decreased significantly. The heatmap showed that the dominant order composition of soil bacteria in N1, N2, and N3 treatments and the CK treatment were different, and the dominant order composition of soil fungi in CK and N3 treatments were different. The N1 treatment showed a significant increase in the Ace and Chao indices of bacteria and Simpson index of fungi. The outcomes of this study suggest that nitrogen addition altered the soil microbial abundance, community structure, and diversity, affecting the soil microbial carbon and nitrogen cycling in permafrost peatland. The results are helpful to understand the microbial mediation on ecological processes in response to N addition.

## 1. Introduction

Nitrogen (N) is the primary restricting factor for plant growth in terrestrial ecosystems [1]. Since the industrial revolution, N deposition has increased significantly [2,3]. The current global N deposition ranges from 0.05 to 2 g N·m^−2^·year^−1^ [4], and it is expected to increase 2.5-fold by the end of the current century [5]. A continuous rise in N deposition has become a global eco-environmental concern [6]. N deposition is the most vital driving factor for carbon sink in China’s terrestrial ecosystem [7]. The aggravation of N deposition has resulted in soil acidification [8]. This, in turn, has altered the composition and diversity of plants and microorganisms [9,10], as well as the soil carbon (C) and N biogeochemical cycles [11], influencing the ecosystem stability. Soil microbes are involved in different biogeochemical cycles, including C and N mineralization [12,13], and they play a crucial role in ecological functioning of the ecosystem. Bacterial and fungal communities are vital components of the soil microbial community and play a crucial role in ecological functioning of ecosystem [14]. The abundance of functional genes encoding key enzymes has gained substantial attention over the recent years. The altered abundance of crucial enzymes could unravel the soil N transformation response mechanism to N addition [15]. Previous studies have shown that N addition could remarkably alter the microbial abundance and community composition by enhancing N availability in the soil, as well as the relationship between above- and belowground ecosystems in peatlands [16,17]. Understanding how microbial abundance and community operate in ecological and biogeochemical processes is essential to understand the mechanisms underlying microbe-driven changes in soil C and N cycling under N addition.

The response of soil microbe to N addition depends on the microbial species, the N treatment duration and amount, and the ecosystem type. The fungal community is more sensitive than the bacterial community to N deposition [18]. A low amount of N addition could increase bacterial and fungal abundance, but a high amount of N addition could decrease bacterial abundance [19]. Mineral N addition decreases soil bacterial diversity, while organic N addition increases soil bacterial diversity [20,21]. As shown previously, short-term N addition increased the richness and Shannon and McIntosh indices of bacterial and fungal communities in the paddy soil [22]. However, long-term N addition decreased bacterial richness by changing soil pH and plant composition in temperate steppe grassland and increased fungal diversity and relative abundance of complex carbohydrate-decomposing bacterial and fungal groups in alpine meadows [23]. Biogeochemical processes and ecosystem functions are directly associated with altered abundance, diversity, and composition of soil microbial communities [18,24]. To predict the responses of ecosystem functions to altered N element levels, it is essential to understand how N addition shapes microbial communities and how these shifts are linked to crucial processes of element cycling. However, the effects of long-term N addition on soil microbial abundance, community composition, and diversity in permafrost peatlands remain ambiguous. Thus, the responses and potential mechanisms of microbial community structure to N addition need to be explored by more field experiments due to limited data and inconsistent responses with other soil.

Most permafrost peatlands are located at high latitudes. These peatlands are often N-limited, due to slow decomposition in cold, acidic, and frequently waterlogged soils, along with more N stored in peatland soil tightly bound with organic matter, compared with other ecosystems [25,26], which are more sensitive to environmental changes. A high climatic temperature promotes soil organic matter mineralization through microbial decomposition, releasing available N that could be absorbed and utilized by plants [27]. The projected 3 °C rise in temperature will increase N mineralization by 7 g N·m^−2^·year^−1^, whereas a 7 °C rise in temperature will increase N mineralization by 9.4 g N·m^−2^·year^−1^ in high-latitude regions [28]. Thus, global warming and N deposition can alleviate the existing N limitation and increase N availability in peatlands [29]. However, only a few studies have explored the response of the soil microbial function and community diversity to altered levels of N elements in permafrost peatland, and the relationship between soil microbial community composition and environmental factors remains unclear. Long-term field studies are scarce, thus hindering determination of the extent and direction of the impact. Thus, it is important to investigate the responses of the soil microbial communities to N addition in permafrost peatlands. In this study, a long-term N addition experiment was performed in the northwest slope of the Great Xing’an Mountains. After nine growing seasons, we collected soil samples and applied real-time PCR (RT-PCR) and high-throughput sequencing techniques to determine the change in the soil microbial functional gene abundance and community diversity under four levels of N addition. RT-PCR was used to detect the abundances of bacteria, fungi, archaea, *nif*H, bacteria-*amo*A (b-*amo*A), *nir*K, *nir*S, *mcr*A, and *pmo*A genes. Illumina Miseq sequencing of bacterial 16S rRNA and fungal ITS genes was used to assess the effect of N addition treatments on the soil bacterial and fungal community composition and diversity. In this study, we focused on two aspects: (1) the response of soil microbial abundance to N addition in N-limited peatland; (2) the relationship of soil substrate properties with the composition and diversity of microbial communities under N addition. We hypothesized that N addition would increase soil microbial abundance and community diversity in permafrost peatland by increasing soil nutrient contents, and that the response would be greater than other soil systems due to stronger N limitation.

## 2. Materials and Methods

### 2.1. Site Description

The study was conducted in a permafrost peatland located at the northwest slope of the Great Xing’an Mountains in Northeast China (52°94′ N, 122°86′ E). The active layer is 50 cm to 60 cm above the permafrost layer. The mean annual air temperature is −3.9 °C, and the mean annual precipitation is 450 mm, with 45% falling as rain from July to August [30]. The dominant plant species are *Eriophorum vaginatum* L., *Vaccinium uliginosum* L., *Chamaedaphne calyculata* L. Moench, *Ledum palustre* L., and *Sphagnum* spp.

### 2.2. Experimental Design and Sampling

The experiment was established in autumn 2011. A total of 12 plots were established; each plot was 2 m × 2 m and separated by a 1 m buffer to prevent increased horizontal movement and lateral loss of N. This experiment entailed a total of four N addition treatments: 0 g N·m^−2^·year^−1^ (CK), 6 g N·m^−2^·year^−1^ (N1), 12 g N·m^−2^·year^−1^ (N2), and 24 g N·m^−2^·year^−1^ (N3); each treatment was applied to three replicated plots. For each N addition treatment, NH_4_NO_3_ was first dissolved in 1 L of surface water and then sprayed evenly on the target area. The same amount of surface water without NH_4_NO_3_ was sprayed in the CK treatment. The treatments were applied during the growing season (from May to September) of 2012 to 2020. The soil samples were sampled from 0–20 cm below the plant litter layer from each plot on 13 August 2020, and five sampling points were collected with a soil drill from each plot and mixed to form a composite sample. The soil samples were divided into subsamples for further analysis. One of the subsamples was stored at −80 °C to determine the functional gene abundance and community diversity of soil microbes. The second soil subsamples were stored at 4 °C for dissolved organic carbon (DOC), ammonia nitrogen (AN), and nitrate nitrogen (NN) content determination. The remaining soil subsamples were air-dried to determine the soil total carbon (TC), total nitrogen (TN), and total phosphorus (TP) content, as well as soil pH.

### 2.3. Microbial Functional Gene Abundance and Community Diversity Analysis

DNA from 0.3 g of soil samples was extracted using a FastDNA^®^Spin Kit for Soil (MPbio, Irvine, CA, USA), according to the manufacturer’s instructions. The extracted DNA was stored at −80 °C prior to functional gene and sequencing analysis. The abundances of bacteria, fungi, archaea, *nif*H, b-*amo*A, *nir*K, *nir*S, *mcr*A, and *pmo*A genes were determined by RT-PCR, which was performed on the ABI StepOne instrument (Applied Biosystems, Beverly, MA, USA) and SYBR green dye. RT-PCR analysis for each soil sample was replicated three times. RT-PCR primers for target gene amplification and the detailed procedure are presented in Table S1. The 25 µL PCR reaction mixture contained 12.5 µL of SYBR Buffer (TaKaRa, Beijing, China), 0.4 µL of each primer (10 µM), 0.5 µL of ROXII (TaKaRa, Beijing, China), 0.88 µL of 3% BSA, 0.63 µL of DMSO, and 10 ng of template DNA. For standard curve generation, the amplicon products of phylogenetic and functional markers were purified using a Cyclic Purification Kit (OMEGA Bio-Tek, Norcross, GA, USA), ligated to the vector pMD18-T (TaKaRa, Beijing, China), and then transformed into TOP10 *Escherichia coli* competent cells. The plasmids were extracted using the Plasmid Mini Kit (OMEGA Bio-Tek, USA/Georgia). The specificity of plasmids was determined through the Basic Local Alignment Search Tool [31], and the plasmid concentration was determined using a Nanodrop 2000 (Thermo, Waltham, MA, USA). The standard curve was obtained by continuous dilution of known copy number plasmids.

For microbial community analyses, the V3–V4 region of the 16S rDNA gene was amplified with primers 338f (5′–ACTCCTACGGGAGGCAGCAG–3′) and 806r (5′–GGACTACHVGGGTWTCTAAT–3′) [32], and the ITS1 region of the 18S rDNA gene was amplified with primers ITS1f (5′–CTTGGTCATTTAGAGGAAGTAA-3′) and ITS2r (5′-GCTGCGTTCTTCATCGATGC–3′) [33] under the following conditions: initial denaturation at 95 °C for 3 min, followed by 35 cycles of 95 °C for 30 s, 55 °C for 30 s, and 72 °C for 45 s, and a final extension at 72 °C for 10 min. The PCR products were purified using a Qia quick PCR Purification kit (Qiagen, Dusseldorf, Germany). The PCR products of different samples were pooled and sequenced on the Illumina MiSeq platform (Illumina, San Diego, CA, USA). Raw sequences of different samples were separated using barcodes, and up to one mismatch was allowed while using the FLASH tool [34]. The quality of the sequence was strictly filtered using the QIIME tool, and Btrim [35] was employed for quality trimming and removing the low-quality regions (Q < 20), the sequences < 150 bp in length, and singletons. The chimeric sequences were detected and removed using VSEARCH [36] to obtain sequences. OTUs were classified using UCLUST at a 97% similarity level, and singletons were removed. Rarefaction analysis was conducted using the originally detected OTUs [37]. The taxonomic assignment was conducted using the Ribosomal Database Project (RDP) classifier [38] with minimal 80% confidence estimates. The RDP classifier was used to assign taxonomic data to each representative sequence. All sequences from this study were deposited at NCBI with accession number PRJNA735903.

### 2.4. Soil Chemical Analysis

The soil total carbon (TC) content was determined via the dry combustion method with the multi N/C 2100 analyzer (Analytik Jena, Jena, Germany). Dissolved organic carbon (DOC) in the soil was determined, as per the method described by Ghani et al. [39]. Total N (TN) and total phosphorus (TP) in the soil samples were digested using sulfuric acid and later quantified through an AA-3 continuous flow analyzer (Seal Analytical, Germany/Norderstedt). Soil ammonium N (AN) and nitrate N (NN) were extracted with 2 mol·L^−1^ KCl and then analyzed with the AA-3 continuous flow analyzer (Seal Analytical, Norderstedt, Germany). Soil pH values were analyzed in a 5:1 water–soil solution.

### 2.5. Statistical Analyses

Data were analyzed using SPSS software (v. 16.0, Chicago, IL, USA) with an accepted significance level of α = 0.05. One-way analysis of variance (ANOVA) and a post hoc Duncan’s multiple-range test were performed to determine the significant differences between the soil properties from different N addition treatments. A nonmetric multidimensional scaling (NMDS) ordination to illustrate the clustering of bacterial and fungal community composition variation was conducted on the Bray–Curtis distance of the order. Pearson’s correlation coefficients between diversity indices and soil microbial functional group abundance, as well as soil microbial community diversity indices, were calculated. Redundancy analyses (RDA) were conducted on soil chemical properties with microbial diversity; specific bacteria and fungi were analyzed separately. RDA analyses were conducted with CANOCO 5.0 software (Beijing, China).

## 3. Results

### 3.1. Soil Chemical Properties

Soil TC content was found to be in the range of 388.57 mg·g^−1^ to 406.53 mg·g^−1^. TC content was lower in the N3 treatment than in N1, N2, and CK treatments. DOC content in all treatments ranged from 331.00 mg·kg^−1^ to 566.47 mg·kg^−1^. DOC content in the N1 treatment was higher than in CK, N2, and N3 treatments (Table 1). TN content ranged from 14.07 mg·g^−1^ to 24.70 mg·g^−1^ in the all treatments, and TN in N1 and N3 treatments was lower than in the N2 treatment. Furthermore, AN content ranged from 39.94 mg·kg^−1^ to 123.41 mg·kg^−1^, and NN content ranged from 34.01 mg·kg^−1^ to 55.06 mg·kg^−1^ in all treatments. AN was higher in the N1 treatment than in CK, N2, and N3 treatments. Furthermore, NN was higher in N1 and N2 treatments than in CK and N3 treatments. TP content ranged from 1.41 mg·g^−1^ to 1.98 mg·g^−1^ in all treatments. TP decreased significantly with the increase in N addition (Table 1). Soil pH values ranged from 5.73 to 5.83, and they did not vary significantly with the addition of different amounts of N (Table 1).

### 3.2. Soil Microbial Functional Group Abundance

**Figure 1:**
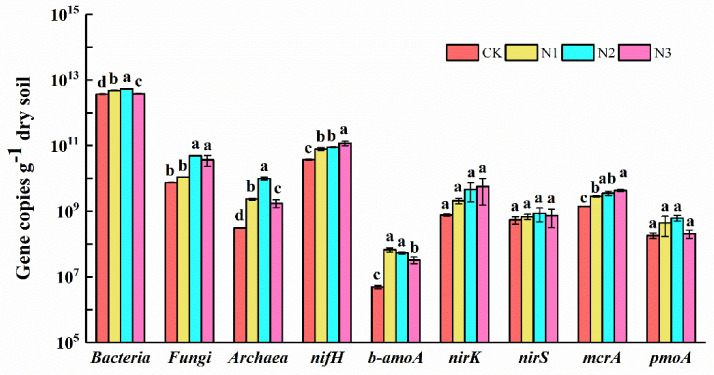
The effect of nitrogen addition on soil microbial functional gene abundance in permafrost peatland. CK: 0 g N·m^−2^·year^−1^; N1: 6 g N·m^−2^·year^−1^; N2: 12 g N·m^−2^·year^−1^; N3: 24 g N·m^−2^·year^−1^. Different letters indicate a significant difference among different concentrations of nitrogen addition treatments (*p* < 0.05) as estimated by one-way ANOVA and a post hoc Duncan’s multiple-range test.

Abundances of bacteria, fungi, archaea, *nif*H, b-*amo*A, denitrification genes (*nir*K, *nir*S), *mcr*A, and *pmo*A were determined using RT-PCR analysis. In all four N addition treatments, bacterial abundance (3.71 × 10^12^ copies·g^−1^ to 5.36 × 10^12^ copies·g^−1^ dry soil) was more higher than fungal abundance (7.44 × 10^9^ copies·g^−1^ to 4.92 × 10^10^ copies·g^−1^ dry soil) and archaeal abundance (3.10 × 10^8^ copies·g^−1^ to 9.92 × 10^9^ copies·g^−1^ dry soil) (Figure 1). Compared with CK, the abundances of bacteria, fungi, and archaea were higher in N addition treatments and highest in the N2 treatment. In addition, the abundances of *nif*H, b-*amo*A and *mcr*A were higher in N addition treatment groups than in CK (Figure 1). The abundance of *nir*K tended to increase with the increase in N addition level, but this increase was not significant (Figure 1).

### 3.3. Soil Microbial Community Composition and Diversity

**Figure 2:**
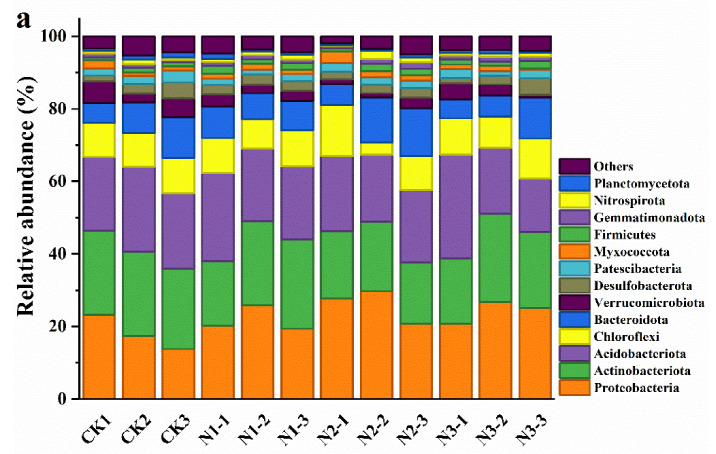

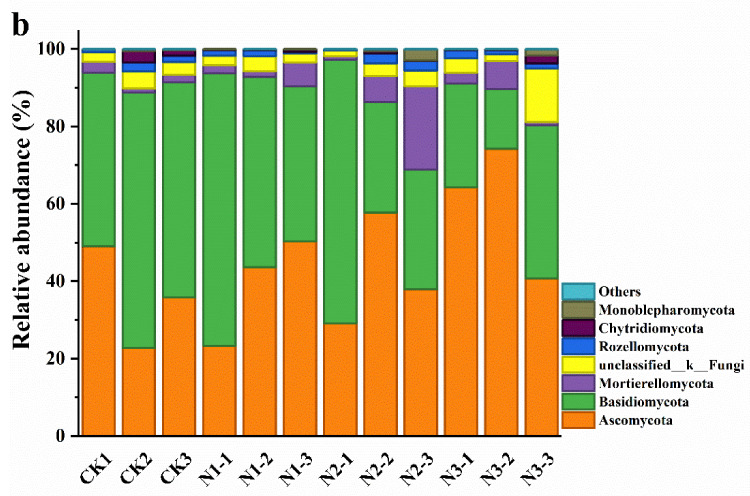
The effect of nitrogen addition on relative abundances of (**a**) bacteria phyla and (**b**) fungi phyla. Displayed are phyla with >1% relative abundance. Unclassified bacterial and fungal phyla < 1% abundance were grouped as “others”. CK1, CK2, CK3: 0 g N·m^−2^·year^−1^; N1-1, N1-2, N1-3: 6 g N·m^−2^·year^−1^; N2-1, N2-2, N2-3: 12 g N·m^−2^·year^−1^; N3-1, N3-2, N3-3: 24 g N·m^−2^·year^−1^.

In the CK plot, the dominant bacterial phyla were Actinobacteria (22.81%) and Acidobacteria (21.45%), followed by Proteobacteria (18.05%), Chloroflexi (9.6%), Bacteroidota (8.26%), Verrucomicrobiota (4.62%), Desulfobacterota (2.88%), Patescibacteria (2.52%), Myxococcota (1.44%), Firmicutes (1.04%), Gemmatimonadota (0.91%), Nitrospirota (0.88%), and Planctomycetota (1.14%) (Figure 2a). Actinobacteria was the most dominant bacterial phylum in all N addition treatments. The Proteobacteria relative abundance in the N2 treatment was significantly higher than that in N1 and N3 treatments. However, the relative abundances of Actinobacteria and Verrucifera in the N2 treatment and Patescibacteria in the N1 treatment were significantly lower than in the N3 treatment (Figure 2a). In the CK plot, the dominant fungal community was Ascomycota (49.89%), followed by Basidiomycota (44.31%), Mortierellomycota (1.41%), unclassified-k-Fungi (2.91%), Rozellomycota (1.16%), Chytridiomycota (1.06%), and Monoblepharomycota (0.21%). Ascomycota was the most dominant fungal phylum in all N addition treatments. The abundance of Basidiomycota decreased, while the abundances of Mortierellomycota and Rozellomycota increased with the increase in N addition amount. However, no significant differences were observed across the fungal phyla in different N addition treatments (Figure 2b).

**Figure 3:**
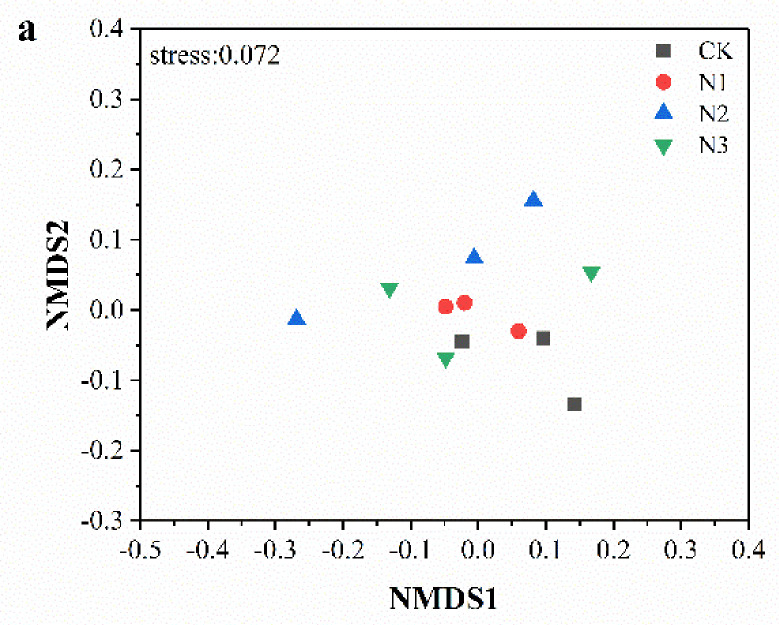

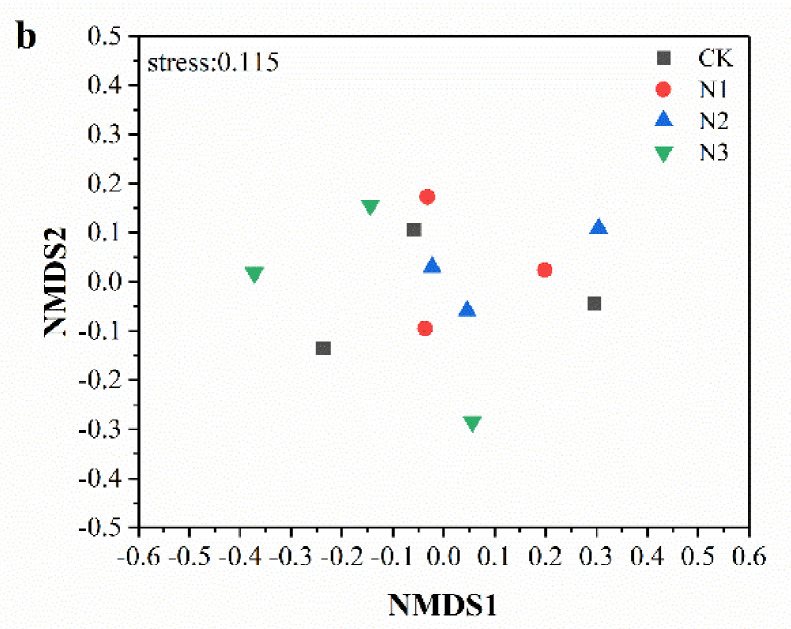
NMDS ordination based on Bray–Curtis similarities of (**a**) bacterial and (**b**) fungal communities under nitrogen addition. CK: 0 g N·m^−2^·year^−1^; N1: 6 g N·m^−2^·year^−1^; N2: 12 g N·m^−2^·year^−1^; N3: 24 g N·m^−2^·year^−1^.

**Figure 4:**
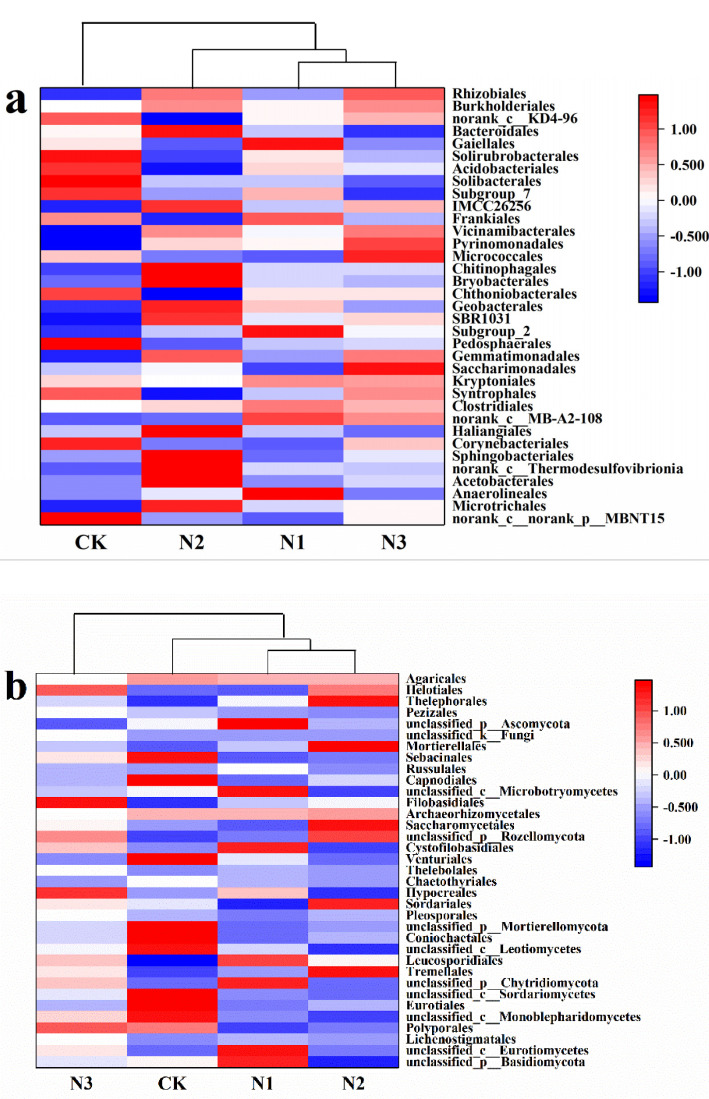
The relative abundances of (**a**) bacteria and (**b**) fungi order with the first 35 most abundant genera were made into a clustering heat map. CK: 0 g N·m^−2^·year^−1^; N1: 6 g N·m^−2^·year^−1^; N2: 12 g N·m^−2^·year^−1^; N3: 24 g N·m^−2^·year^−1^.

NMDS analysis based on Bray–Curtis similarity distance of order showed that the composition of soil bacteria in N1, N2, and N3 treatments clustered more closely together than in the CK treatment (Figure 3a), and fungal composition in CK, N1, and N2 treatments clustered more closely together (Figure 3b). The heatmap graphically showed that the order of soil bacterial and fungal communities differed with N addition treatment, which supported the NMDS analyses (Figure 4). We observed that the composition of soil bacterial dominant order in N1, N2, N3, and CK treatments were different. The relative abundances of Solibacterales, Pedosphaerales, and Corynebacteriales were highest in the CK treatment and lowest in N3, N2, and N1 treatments, respectively; there was a significant difference in abundance between CK and different N addition treatments (*p* < 0.05) (Figure 4a). Furthermore, the dominant order composition of soil fungi was different between CK and N3 treatments. Compared with the CK treatment, the relative abundance of unclassified_p_Mortierellomycota, Coniochaetales, and unclassified_c_Monoblepharidomycetes showed significant differences under N3 treatment (*p* < 0.01) (Figure 4b).

**Figure 5:**
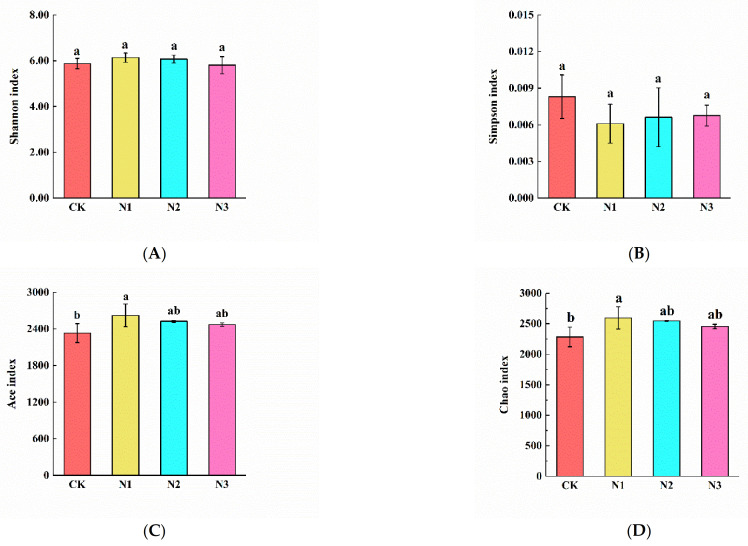
The alpha diversity of soil bacteria under nitrogen addition treatment. CK: 0 g N·m^−2^·year^−1^; N1: 6 g N·m^−2^·year^−1^; N2: 12 g N·m^−2^·year^−1^; N3: 24 g N·m^−2^·year^−1^. (**A**): Shannon index; (**B**): Simpson index; (**C**): Ace index; (**D**): Chao index. Different letters indicate a significant difference among different concentrations of nitrogen addition treatments (*p* < 0.05) as estimated by one-way ANOVA and the subsequent Duncan’s multiple-range test.

**Figure 6:**
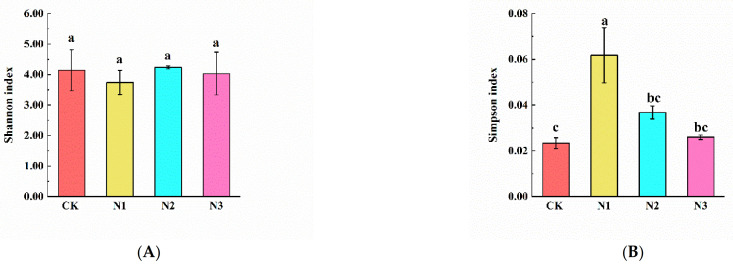

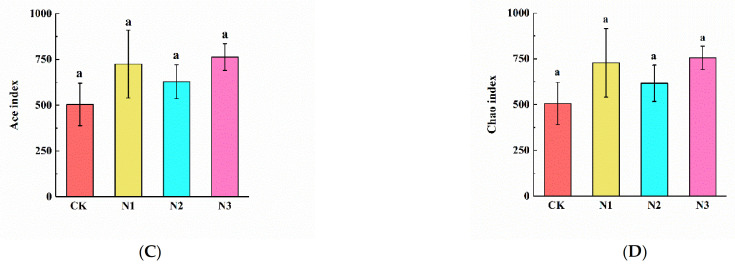
The alpha diversity of soil fungi under nitrogen addition treatment. CK: 0 g N·m^−2^·year^−1^; N1: 6 g N·m^−2^·year^−1^; N2: 12 g N·m^−2^·year^−1^; N3: 24 g N·m^−2^·year^−1^. (**A**): Shannon index; (**B**): Simpson index; (**C**): Ace index; (**D**): Chao index. Different letters indicate a significant difference among different concentrations of nitrogen addition treatments (*p* < 0.05) as estimated by one-way ANOVA and a post hoc Duncan’s multiple-range test.

The Shannon, Simpson, Ace, and Chao indices were used to estimate and compare the alpha diversity of bacterial and fungal communities in N1, N2, and N3. We observed that Ace and Chao indices of bacteria in N1 treatment were significantly higher than in CK and differed significantly between N1 and CK treatments (Figure 5C,D). The Simpson index of fungi in the N1 treatment was significantly higher than in CK, N2, and N3 treatments (Figure 6B).

### 3.4. The Relationship between Soil Microorganisms and Soil Chemical Properties under Nitrogen Addition

**Figure 7:**
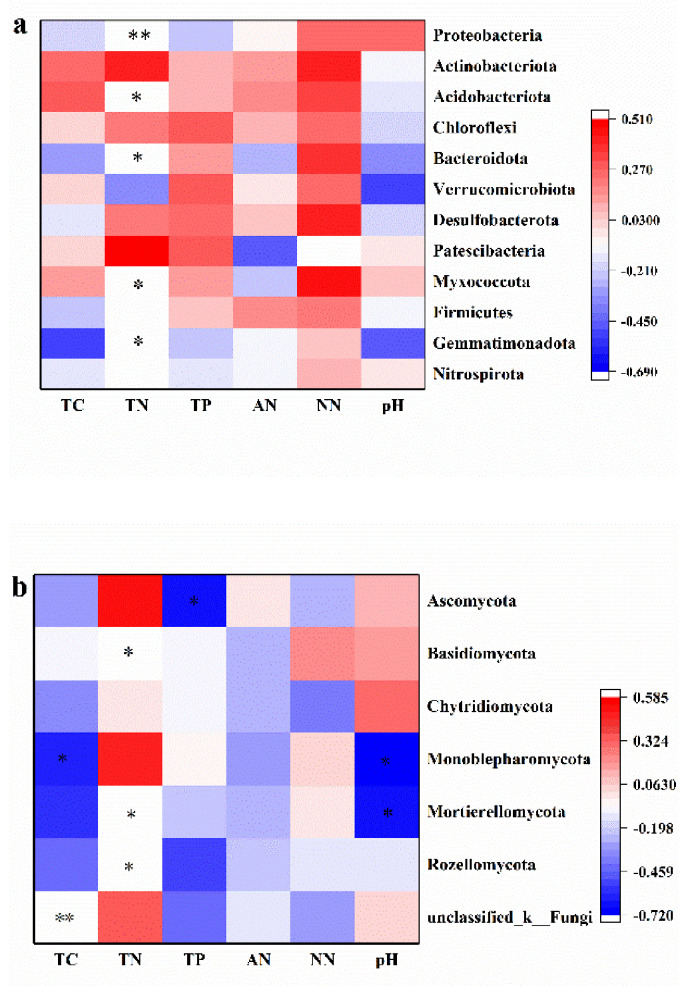
Correlation analysis of soil microbial relative abundance at (**a**) bacteria and (**b**) fungi phyla level and soil chemical properties under nitrogen addition. * significant at 0.05 level, ** significant at 0.01 level.

Correlation analysis of microbial abundance and soil chemical properties showed that the abundances of bacteria, fungi, archaea, and b-*amo*A were significantly and positively correlated to TN. The abundance of b-*amo*A was significantly and positively correlated to AN (Table 2). Moreover, the relative abundances of Proteobacteria (*r* = 0.792, *p* < 0.01), Actinobacteria (*r* = 0.589, *p* < 0.05), Bacteroidota (*r* = 0.659, *p* < 0.05), Myxococcota (*r* = 0.620, *p* < 0.05), and Gemmatimonadota (r = 0.668, *p* < 0.05) were positively correlated to TN (Figure 7a). The relative abundance of Ascomycota was negatively correlated to TP (*r* = −0.674, *p* < 0.05). The relative abundances of Basidiomycota (*r* = 0.696, *p* < 0.05), Mortierellomycota (*r* = 0.605, *p* < 0.05), and Rozellomycota (*r* = 0.687, *p* < 0.05) were positively correlated to TN. The relative abundance of Mortierellomycota was negatively correlated to pH (*r* = −0.673, *p* < 0.05). The relative abundance of unclassified-k-Fungi was negatively correlated to TC (*r* = −0.787, *p* < 0.01). The relative abundance of Monoblepharomycota was negatively correlated to TC and pH (*r* = −0.590, *p* < 0.05; *r* = −0.673, *p* < 0.05) (Figure 7b).

**Table 2:** Pearson correlation analysis of soil microbial abundance and soil chemical properties. TC: total carbon; TN: total nitrogen; TP: total phosphorus; AN: ammonium nitrogen; NN: nitrate nitrogen; DOC: dissolved organic carbon. * significant at the 0.05 level, ** significant at the 0.01 level. Bold text indicates statistically significant correlations.

Indicator	Bacteria	Fungi	Archaea	*nif*H	B-*amoA*	*nir*K	*nir*S	*mcr*A	*pmo*A	TC	TN	TP	AN	NN	DOC	pH
Bacteria	1															
Fungi	0.42	1														
Archaea	0.85 **	0.75 **	1													
*nif*H	0.17	0.68 *	0.30	1												
B-*amoA*	0.75 **	0.31	0.51	0.46	1											
*nir*K	0.14	0.73 **	0.36	0.73 **	0.27	1										
*nir*S	0.31	0.48	0.35	0.44	0.28	0.78 **	1									
*mcr*A	0.26	0.73 **	0.41	0.94 **	0.48	0.74 **	0.47	1								
*pmo*A	0.81 **	0.42	0.65 *	0.19	0.61 *	0.13	0.21	0.22	1							
TC	−0.05	−0.36	−0.24	−0.45	0.11	−0.35	−0.25	−0.55	0.11	1						
TN	0.86 **	0.75 **	0.92 **	0.36	0.63 *	0.35	0.42	0.47	0.70	−0.13	1					
TP	0.08	−0.52	−0.09	−0.55	−0.25	−0.44	−0.20	−0.26	0.34	0.28	−0.28	1				
AN	−0.01	−0.17	−0.30	0.43	0.58 *	0.10	−0.02	0.36	−0.04	0.22	−0.09	−0.32	1			
NN	0.49	−0.10	0.33	−0.26	0.40	−0.22	0.10	−0.28	0.19	0.18	039	0.13	0.02	1		
DOC	0.02	−0.44	−0.35	−0.19	0.37	−0.10	0.11	−0.67	0.07	0.74 **	0.18	0.23	0.56	0.36	1	
pH	−0.18	0.03	−0.17	0.18	0.04	−0.38	−0.53	0.04	0.04	0.12	−0.04	−0.30	0.15	−0.13	−0.13	1

**Figure 8:**
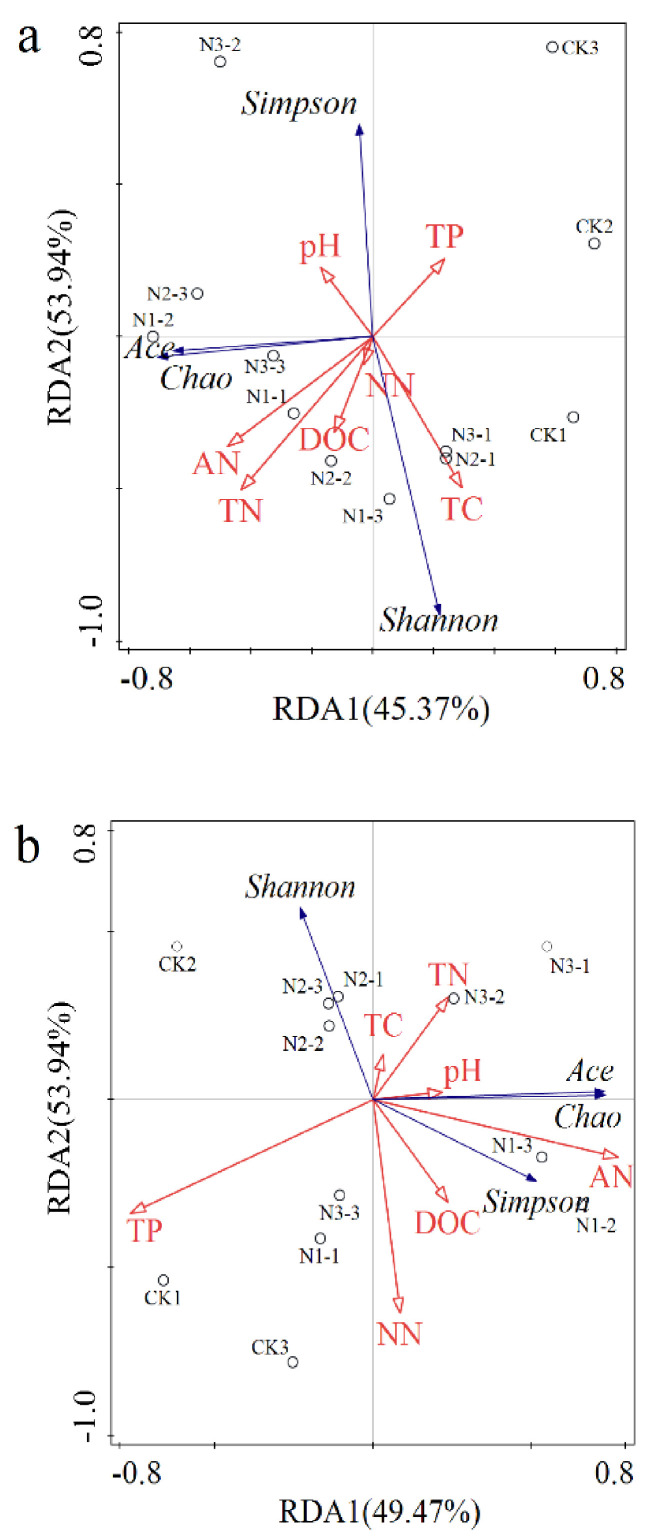
Redundancy analysis (RDA) of soil chemical properties and the alpha diversity indices of (**a**) bacteria and (**b**) fungi microbial communities under nitrogen addition treatments. TC: total carbon; TN: total nitrogen; TP: total phosphorus; AN: ammonium nitrogen; NN: nitrate nitrogen; DOC: dissolved organic carbon. CK1, CK2, CK3: 0 g N·m^−2^·year^−1^; N1-1, N1-2, N1-3: 6 g N·m^−2^·year^−1^; N2-1, N2-2, N2-3: 12 g N·m^−2^·year^−1^; N3-1, N3-2, N3-3: 24 g N·m^−2^·year^−1^. The red arrows and blue arrows represent soil chemical properties and the alpha diversity indices of bacteria and fungi microbial communities, respectively.

The RDA of soil chemical properties and alpha diversity indices of microbial community in all N treatments is shown in Figure 8. The Shannon index of bacteria was positively correlated to TC, TN, DOC, AN, and NN; however, the Simpson index of bacteria was negatively correlated to TC, TN, DOC, AN, and NN. The Shannon index of bacteria was negatively correlated to TP and pH, and the Simpson index of bacteria positively correlated to TP and pH. Ace and Chao indices of bacteria were positively correlated to TN, DOC, AN, pH, and NN, but negatively correlated to TC and TP. With 23.9%, 20.6%, and 15.4% contribution rates, AN, TN, and TC contributed remarkably to bacterial diversity, respectively (Figure 8a). The Shannon index of fungi was positively correlated to TC and TN, but negatively correlated to AN, NN, DOC, and pH. Simpson, Ace, and Chao indices of fungi were positively correlated to AN, NN, DOC, and pH, but negatively correlated to TP. The contribution rates of AN and TP to fungal diversity indices were 56.7% and 29.3%, respectively (Figure 8b).

## 4. Discussion

### 4.1. Effect of N Addition on Soil Microbial Functional Gene Abundance in Permafrost Peatland

Soil microbes contribute significantly to all the biogeochemical processes of soil [12,13], such as soil carbon mineralization, methane production and oxidation, N fixation, and nitrification or denitrification [40]. The change of nutrient environment can affect the key microbes related to soil C and N cycles in different directions and to different degrees. Our observations supported our hypothesis that the microbial abundance in the soil of permafrost peatlands increased under N addition. Compared with control, the abundances of bacteria, fungi, archaea, *nif*H, b-*amo*A, and *mcr*A were higher in N addition treatments. In line with our hypothesis, soil *nif*H (increased 110% to 213%) and b-*amo*A (increased 567% to 1262%) abundances in permafrost peatland were more sensitive to N addition than agricultural soils (*nif*H: no effect; b-*amo*A: increased 313%) according to a meta-analysis result of 47 field studies [41]. However, contrary to our hypothesis, soil *nir*K (no effect) and *nir*S (no effect) genes were less sensitive to N addition in permafrost peatland than in agricultural soils, increasing 53% and 40%, respectively [41]. The abundances of bacteria, fungi, archaea, b-*amo*A, and *mcr*A were found to increase under N addition, indicating that N addition may promote microbial decomposition of soil organic matter, ammonia oxidation, N fixation, and methane production. Previous studies have also demonstrated that the abundances of bacteria, fungi, archaea [42], and b-*amo*A [43] increased with N addition. Orr et al. [44] showed that N addition increased the abundance of N-fixing bacteria and promoted the N-fixing function of soil microbes in cropland. In line with the findings of our study, N addition induced an increase in abundance of *nif*H in Antarctic soils [45]. The response of *nif*H to different levels of N addition was different, related to the cultivar and the amount of added N [46]. As reported previously, Juraeva et al. [47] found that the relative abundance of the *nif*H gene pool in plant roots was positively correlated with N supply. Coelho et al. [48] also found that a low concentration of N input could improve the *nif*H gene abundance in the rhizosphere of sorghum bicolor. We previously found that N addition could increase the biomass of plant roots in the same N addition plots [49]. Therefore, N addition could improve *nif*H abundance by promoting plant root growth and secreting more exudates. In contrast, Tian et al. [50] demonstrated that N addition decreased the abundance of N-fixing bacteria in forest ecosystems. N addition did not have a significant effect on *nir*K gene abundance in permafrost peatland soil. Previous researchers have also obtained consistent results suggesting that nitrite reductase (NO-forming) is insensitive to environmental changes [51]. Wang et al. [18] reported that N addition induced a decrease in the abundances of soil bacteria and fungi in subtropical forest, which might have been associated with the significant decrease in soil pH. A lower soil pH could trigger aluminum toxicity, hampering microbial growth [52]. However, in the current study, as N addition did not induce significant soil acidification, we concluded that pH may be not the major regulatory factor for soil microbial abundance in permafrost peatlands.

### 4.2. Effects of N Addition on Soil Microbial Community Composition and Diversity in Permafrost Peatland

The community structure and diversity of soil microorganisms respond distinctly to different N addition levels [53]. At the phylum level, the relative abundances of bacteria showed different trends in response to different N addition levels. N addition could alter microbial community composition [54], resulting in the altered relative abundance of specific bacteria phyla [55]. Compared with CK, N1, and N3 treatments, the relative abundance of Proteobacteria in the N2 treatment was highest. Proteobacteria, which comprise eutrophic bacteria, grow and reproduce rapidly in high-N environments [16]. Moreover, N addition decreased the relative abundances of Acidobacteria and Verrucifera. Furthermore, the relative abundances of Actinobacteria and Verrucifera in the N2 treatment were significantly lower than in CK. In line with the findings of our study, N addition induced a decrease in the abundances of Actinobacteria and Verrucifera in the topsoil of subtropical acidic forests [56]. The plausible reason might be that Actinobacteria and Verrucifera belong to the oligotrophic group, and their lower growth rate makes them more suitable for soil conditions with lower nutrient levels [16]. Additionally, these microbes have a lower ability to use carbon sources in a high-N environment [57]. We observed that Ascomycota was the most dominant fungal phylum. In line with a previous study by Zhu et al. [58], the abundance of Basidiomycota decreased, whereas the abundances of Mortierellomycota and Rozellomycota increased with the N addition amount. The soil bacterial dominant orders in different N addition and CK treatments were different, while the dominant order composition of soil fungi in the CK treatment was different to that in the N3 treatment. The relative increases or decreases in abundance of soil microorganisms with the availability of nitrogen may be due to N addition directly or indirectly leading to the transformation of lifecycle strategies of major microorganisms; thus, their response to N addition is inconsistent [16,59]. The altered structure and composition of soil microbial community in the N addition reflected the altered nutrient absorption [60].

Consistent with our initial hypothesis, N addition increased the bacterial and fungal diversity, but this depended on the N addition level. We observed that N1 treatment increased the Ace and Chao indices of bacteria and the Simpson index of fungi in permafrost peatlands. Li et al. [23] also reported that N addition increased fungal diversity and differentially affected microbial community composition and structure by modifying microbial preferences in alpine meadows soil. However, these positive effects were different from the results of previous studies, which reported that N addition reduced the bacterial Chao1 index in agricultural grassland and forests soils [56,61] and the fungal Simpson index in forests soils [62]. Furthermore, as reported previously, N addition decreased bacterial richness by decreasing soil pH; thus, N addition negatively affected soil microbial communities in forest soils [18,63]. However, in the current study, the soil pH did not change significantly, suggesting that the altered soil microbial diversity was closely correlated with soil nutrients, but little correlated with soil pH under N addition in permafrost peatlands. Under the condition of long-term N addition, the composition of the soil microbial community may be altered, but the soil pH did not change significantly [64,65]. The results showed that the responses of the Simpson index of fungi to N addition were more sensitive than those of the Simpson index of bacteria. This finding is in line with Yang et al. [66], who showed that soil fungal diversity was more sensitive than soil bacterial diversity under global N addition, because soil fungi had a higher carbon and nutrient assimilation efficiency than soil bacteria.

### 4.3. Effects of Soil Chemical Properties on the Soil Microorganisms under Nitrogen Addition in Permafrost Peatland

N addition could alter soil properties directly or indirectly by affecting soil microbial communities [63]. We observed that DOC decreased in N2 and N3 treatments, which might have been due to N addition-induced increase in the soil labile carbon decomposition [28,67]. High levels of N addition decreased soil DOC concentrations, indicating that soil active organic C, an energy source for microbial growth, responded to N addition. These changes are most likely because N addition stimulated the growth of microorganisms and changed C-use efficiency due to the exhaustion of active C substrates [68]. Fang et al. [69] reported that the decrease in DOC concentration in soil was a consequence of changing microbial decomposition and humidification processes. Soil TP content significantly decreased in the N3 treatment due to the increase in absorption and utilization of TP by plants in a high-N environment [28,67], limiting P availability compared to N [23]. The integrated effect of soil organic carbon stimulation resulted in no significant effect on TC in different N addition treatments. N addition promoted the decomposition of active organic C. On the other hand, N addition provided more N nutrition for plant growth, improved plant productivity, and increased the carbon input of plant litter, resulting in an unclear change in soil TC content [68]. However, current studies on soil carbon sequestration potential under N addition-driven conditions are divergent [70]. N input significantly stimulated an increase in soil carbon level in some ecosystems [71] but a significant decrease in others [72] or remained constant [73], depending on ecosystem types and N addition level.

We analyzed soil microbial functional gene abundance and diversity along with soil chemical properties in different N addition treatments. The outcomes suggested that bacterial, archaeal, and b-*amo*A abundances were closely related to TN, and that soil N is a major factor regulating the abundances of soil bacteria, archaea, and b-*amo*A [74,75]. We also observed that Proteobacteria, Actinobacteria, Bacteroidota, Myxococcota, Gemmatimonadota, Basidiomycota, Mortierellomycota, and Rozellomycota were correlated to TN, whereas Ascomycota was correlated to TP. This indicates that N addition influenced soil microbial community structure by altering soil nutrient level.

## 5. Conclusions

This study demonstrated that continued N addition for nine growing seasons increased the abundances of bacteria, fungi, archaea, *nif*H, b-*amo*A, and *mcr*A in permafrost peatland, indicating that N addition could stimulate the decomposition of soil organic matter, oxidation of ammonia, fixation of N, nitrification, and production of methane, thereby affecting soil C and N cycling. Soil *nif*H and b-*amo*A abundances in permafrost were more sensitive to N addition than agricultural soils, but *nir*K and *nir*S abundances were less sensitive to N addition in peatland soil than in agricultural soils. N3 treatment changed the dominant order composition of soil fungi. In addition, N1 treatment increased the Ace and Chao indices of bacteria and the Simpson index of fungi. The positive effects of N addition on diversity indices of soil bacteria and fungi were different from grassland and forests soils, indicating that soil microbial diversity was closely correlated with soil nutrients but not soil pH under N addition in permafrost peatlands. The results highlight the important role of microbes in regulating soil ecological processes in permafrost peatlands under N addition. This study focused only on the effects of N addition on belowground microbial processes; thus, future studies should explore the interaction and response mechanism of aboveground plants and soil microbes to N addition.

## Figures and Tables

**Table 1:** Effects of nitrogen addition on the content of soil carbon, nitrogen and phosphorus. Values are means ± standard error (*n* = 3). TC: total carbon; TN: total nitrogen; TP: total phosphorus; AN: ammonium nitrogen; NN: nitrate nitrogen; DOC: dissolved organic carbon. CK: 0 g N·m^−2^·year^−1^; N1: 6 g N·m^−2^·year^−1^; N2: 12 g N·m^−2^·year^−1^; N3: 24 g N·m^−2^·year^−1^. Different letters indicate a significant difference among different concentrations of nitrogen addition treatments (*p* < 0.05) as estimated by one-way ANOVA and a post hoc Duncan’s multiple-range test.

	TC (mg·g^−1^)	TN (mg·g^−1^)	TP (mg·g^−1^)	AN (mg·kg^−1^)	NN (mg·kg^−1^)	DOC (mg·kg^−1^)	pH
CK	403.23 ± 12.77a	14.07 ± 1.08c	1.98 ± 0.01a	39.94 ± 9.48c	45.75 ± 17.22ab	421.53 ± 91.65ab	5.73 ± 0.02a
N1	406.53 ± 2.53a	18.28 ± 0.62b	1.79 ± 0.29ab	123.41 ± 1.13a	55.06 ± 0.82a	566.47 ± 63.89a	5.76 ± 0.02a
N2	392.89 ± 10.55a	24.70 ± 1.09a	1.70 ± 0.12ab	46.56 ± 5.76c	53.82 ± 2.44ab	331.00 ± 15.51b	5.75 ± 0.20a
N3	388.57 ± 9.92a	16.90 ± 1.22b	1.41 ± 0.20b	95.81 ± 10.57b	34.01 ± 0.19b	351.33 ± 87.57b	5.83 ± 0.06a

## Data Availability

Data are available on request.

## Supplementary Materials

The following are available online at https://www.mdpi.com/article/10.3390/microorganisms9122498/s1, **Table S1**: PCR primers and amplification details used for the amplification of functional targets.
