# Supplementary material for: Effect of Nitrogen Addition on Soil Microbial Functional Gene Abundance and Community Diversity in Permafrost Peatland
Source: Microorganisms. 2021 Dec 2;9(12):2498. doi: 10.3390/microorganisms9122498 (PMC8707234; doi:10.3390/microorganisms9122498)
Supplement: Supplementary file 1 [file microorganisms-09-02498-s001.zip › microorganisms-1462068-SI.pdf]

Table S1 PCR primers and amplification details used for the amplification of functional target

Target group	Primer	Reference	Sequence (5′—3′)	Amplification details
Bacteria	Bacteria-338F<br>Bacteria-518R	[1]	CCTACGGGAGGCAGCAG<br>ATTACCGCGGCTGCTGG	95°C 2min, 35 cycles, 95°C 30s, 60°C 30s,72°C 30s, 80°C 15s
Fungi	ITSIF<br>5.8S	[2]	TCCGTAGGTGAACCTGCGG<br>CGCTGCGTTCTTCATCG	94 °C 15min, 94 °C 30s, 59.4 °C 30s, 72 °C 30s, 80°C 30s, 35 cycles
Archaea	Ar-109F<br>Ar-912R	[3]	ACGGCTCAGTAACACGT<br>CTCCCCCGCCAATTCCTTTA	95 °C 2min, 95 °C 30s, 57 °C 30s, 72 °C 30s, 80°C 30s, 35 cycles
*Bacterial-nif*H	PolF<br>PolR	[4]	TGCCAYCCSAARGCBGACTC<br>ATSGCCATCATYTCRCCGGA	95°C 10min, 40 cycles, 95°C 15s, 60°C 30s,72°C 30s, 80°C 15s
*Bacterial-amo*A	amoA1F<br>amoA2R	[5]	GGGGTTTCTACTGGTGGT<br>CCTCKGSAAAGCCTTCTTC	95 °C 5min, 95 °C 30s, 55 °C 30s,72 °C 30s, 83 °C 15s,40 cycles
*Bacterial-nir*K	F1aCu<br>R3Cu	[6]	ATCATGGTSCTGCCGCG<br>GCCTCGATCAGRTTGTGGTT	95 °C 10min, 6 touch down cycles: 95 °C 15s, 63 °C 30s (-1 °C), 72 °C 30s; 95 °C 15s, 58 °C 30s, 72 °C 30s, 80 °C 30s, 35cycles
*Bacterial-nir*S	cd3aF<br>R3cd	[7]	GTSAACGTSAAGGARACSGG<br>GASTTCGGRTGSGTCTTGA	95 °C 10min, 94 °C 1min, 57 °C 1min, 72 °C 1min, 83°C 30s, 40 cycles
*mcr*A	mlas<br>mcrA-rev	[8]	GGTGGTGTMGGDTTCACMCARTA<br>CGTTCATBGCGTTVGGRTAGT	95℃ 3min 30s, 36 cycles, 95℃ 15s, 55℃ 30s, 72℃ 30s, 83℃ 30s
*pmo*A	A189f<br>mb661r_nd	[9]	GGNGACTGGGACTTCTGG<br>CCGGMGCAACGTCYTTACC	95℃ 10min, 40cycles, 95℃ 30s, 60℃ 30s, 72℃ 30s, 85℃ 15s

1. Wang, H.; Yang, J.P.; Yang, S.H.; Yang, Z.C.; Lv, I.M. Effect of a 10 degrees C-elevated temperature under different water contents on the microbial community in a tea orchard soil. *Eur. J. Soil Biol*. **2014**, *62*, 113-120.
2. Gardes, M.; Bruns, T.D. Its primers with enhanced specificity for basidiomycetes-application of mycorrhizae and rusts. *Microb Ecol*. **1993**, *2* (2), 113-118.
3. Lueders, T.; Friedrich, M. Archaeal population dynamics during sequential reduction processes in rice field soil. *Appl. Environ. Microbiol*. **2000**, *66* (7), 2732-2742.
4. Fan, L. Response of diazotrophic microbial community to nitrogen input and glyphosate application in soils cropped to soybean. *Auburn: Auburn University*. **2013**.
5. Rotthauwe, J.H.; Witzel, K.P.; Liesack, W. The ammonia monooxygenase structural gene amoA as a functional marker: Molecular fine-scale analysis of natural ammonia-oxidizing populations. *Appl. Environ. Microbiol*. **1997**, *63* (12), 4704-4712.
6. Hallin, S.; Lindgren, P.E. PCR detection of genes encoding nitrile reductase in denitrifying bacteria. *Appl. Environ. Microbiol*. **1999**, *65* (4), 1652-1657.
7. Petersen, D.G.; Blazewicz, S.J.; Firestone, M.; Herman, D.J.; Donald, J.; Turetsky, M.; Waldrop, M. Abundance of microbial genes associated with nitrogen cycling as indices

of biogeochemical process rates across a vegetation gradient in Alaska. *Environ. Microbiol.* **2012**, *14* (4), 993-1008.

8. Steinberg, L.M.; Regan, J.M. mcrA-targeted real-time quantitative PCR method to examine methanogen communities. *Appl Environ Microb*. **2009**, 75(13):4435–4442
9. Holmes, A.J.; Roslev, P.; Mcdonald, I.R.; Iversen, N.; Henriksen, K.; Murrell, J.C. Characterization of methanotrophic bacterial populations in soils showing atmospheric methane uptake. *Appl Environ Microb*. **1999**, 65(8):3312–3318
